# Special Considerations in the Management of Women with Epilepsy in Reproductive Years

**DOI:** 10.3390/jpm12010088

**Published:** 2022-01-11

**Authors:** Krishna Parekh, Hannah Debra Kravets, Rebecca Spiegel

**Affiliations:** Department of Neurology, Renaissance School of Medicine at Stony Brook University, Stony Brook, NY 11794, USA; Krishna.Parekh@stonybrookmedicine.edu (K.P.); hannah.kravets@gmail.com (H.D.K.)

**Keywords:** women with epilepsy, catamenial seizures, hormonal changes, hormonal contraceptives

## Abstract

Anti-seizure medications (ASMs) fail to prevent seizure recurrence in more than 30% of patients with epilepsy. The treatment is more difficult in premenopausal women with epilepsy (WWE) because changes in plasma estrogen and progesterone concentrations during the menstrual cycle often affect seizure frequency and intensity. Interactions between enzyme-inducin ASMs and hormonal contraceptives can lead to both a loss of seizure control and failure of contraception. Significant changes in the function of the liver and kidneys during pregnancy can accelerate metabolism and elimination of ASMs, causing breakthrough seizures. In addition, the teratogenic, cognitive, and psychological effects of ASMs on potential offspring have to be considered when choosing the best ASM regimen. Therefore, aspecialized approach is necessary for the treatment of premenopausal WWE.

## 1. Introduction

Fluctuations of plasma estrogen and progesterone levels may lead to the recurrence of seizures in WWE, both during ovulation and the menses; such seizures are known as catamenial seizures [1]. An increase in reproductive disorders in WWE is caused both by the seizures themselves and the effects of the ASMs on the hormonal milieu [2]. Finding the appropriate hormonal contraception can be challenging, due to the complex interactions between these contraceptives and ASMs. Pregnancy further complicates the treatment of WWE due to marked physiologic changes in the liver and kidney function, particularly with respect to ASMs that are metabolized via glucuronidation. These changes mandate careful monthly monitoring of ASM levels to prevent seizure recurrence [3,4,5].

Furthermore, in premenopausal WWE, it is important to choose an ASM that is both effective for the patient’s seizure type and is associated with the lowest risk of teratogenicity and cognitive impairment of a potential offspring. In addition, avoiding polypharmacy when possible and choosing the lowest effective ASM dosage prior to pregnancy have also been proven to be effective in optimizing the health of the offspring [6,7,8]. Lastly, given that the neural tube forms during the first four weeks of gestation, when the woman is not aware of her pregnancy, WWE with childbearing potential are advised to take daily folic acid supplementation during their reproductive years to reduce the risk of neural tube defects and to enhance cognitive development in the offspring [9,10].

## 2. Seizures and Reproductive Health

Seizures can alter levels of hypothalamic and pituitary hormones leading to reproductive endocrine disorders [11,12]. The hypothalamus and pituitary hormones regulating the reproductive system interact with temporolimbic structures that are often involved in the pathogenesis of epilepsy [13]. In women who suffer from both unilateral temporal lobe epilepsy and a reproductive endocrine disorder, the lateralization of epileptiform discharges to the right side was associated with hypogonadotropic hypogonadism, while discharges on the left side were associated with the polycystic ovarian syndrome (PCOS) [13,14].

Studies have also indicated that menstrual disorders are more common in WWE than in the general female population, occurring in one-third of WWE versus 12–14% in the general population [14]. WWE also suffer from PCOS at a higher rate than the general population (10–20% and 5–6%, respectively) [14]. In a retrospective survey study, menstrual disturbances were more frequent among those treated with valproic acid, receiving polytherapy, and those with a higher seizure frequency [15].

Some studies demonstrated that WWE have decreased fertility, compared with the general female population, while others showed no difference [16,17,18,19]. In particular, the WWE Pregnancy Outcomes and Deliveries study revealed no difference in pregnancy rates between WWE and controls (60.7% and 60.2%, respectively) [20]. Pregnancy outcomes were similar between the groups (81.5% having live births); miscarriage rates were also similar (14.8% in WWE and 18.5% in controls), as were sexual activity and ovulatory rates [20].

## 3. Anti-Seizure Medications and Reproductive Health

Enzyme-inducing ASMs can decrease plasma concentrations of reproductive hormones by altering hepatic metabolism [21]. These ASMs can also increase the synthesis of sex hormone-binding globulin further reducing plasma concentration of free estradiol, which overtime may lead to menstrual disorders [22]. Examples of ASMs inducing the changes described above are phenobarbital, phenytoin, and carbamazepine [23]. Valproic acid is a well-known ASM that is implicated in the pathophysiology of PCOS, especially if initiated during early reproductive years [2,14,24,25,26]. Valproic acid causes an increase in plasma androgen levels and induces weight gain, two features associated with PCOS [24,25]. These valproic-induced changes were reversible when valproic acid was switched to lamotrigine in a small study [27].

## 4. Appropriate Anti-Seizure Medications during the Reproductive Age

Ideally, the most appropriate ASM regimen should be selected prior to the woman’s first menses or soon after [6]. The appropriate selection includes choosing a suitable ASM for the patient’s seizure type and avoiding ASMs with teratogenic potential and cognitive side effects [6,28]. The optimal regimen also entails administering ASMs at the lowest effective doses and avoiding polytherapy, when possible [6].

Based on data from pregnancy registries such as the North American, United Kingdom, and Ireland, EURAP, and Australian registries, lamotrigine and levetiracetam are the most prescribed ASMs and have a fairly low teratogenic potential, while valproic acid is associated with the highest risk of major congenital malformations and should be avoided in WWE if possible [8,29,30,31]. Furthermore, along with a significant teratogenic effect, valproic acid was also shown to adversely affect cognition and increase the rate of autism in children who were exposed to this ASM in utero [32,33,34].

Lastly, WWE who are sexually active should receive folic acid supplementation to improve cognition in children exposed to ASMs in utero [6]. The protective effect of folic acid on neural tube defects has not been well established in children exposed to ASMs but has been proven to reduce neural tube defects in the general population and is, thus, recommended for all WWE in the reproductive age group [6,9]. If there is a family history of neural tube defects, carbamazepine and valproic acid should be avoided, as these medications can increase the risk of neural tube defects [35].

## 5. Anti-Seizure Medications and Hormonal Contraceptives

Interactions between ASMs and hormonal contraceptives have major implications in WWE. Hepatically induced ASMs can accelerate the metabolism of estrogen and progesterone, leading to contraceptive failure, unplanned pregnancy, and fetal teratogenicity. At the same time, estrogen and progesterone can reduce the plasma levels of some ASMs thus increasing the risk of seizure recurrence [23].

The increased hepatic metabolism of estrogen and progesterone is most prominent with ASMs that induce cytochrome P450 enzymes (i.e., phenytoin, phenobarbital, and carbamazepine). ASMs that induce the uridine-diphosphate-glucuronosyltransferase enzymes (UGT enzymes) affect the metabolism of estrogen and progesterone to a lesser degree; such ASMs include lamotrigine and oxcarbazepine [21,23]. Topiramate is a special case of a UGT enzyme inducer; this medication has a minimal effect on estrogen and progesterone levels at doses below 200 mg/day but can significantly increase estrogen metabolism at doses above 200 mg/day [36,37].

The table below divides ASMs into the ones that can reduce the effectiveness of oral contraceptives (OCPs) through enzymatic induction and ones that do not. For WWE who are on ASMs that can affect OCP levels, it is best, if at all possible, to avoid OCPs and use either a barrier method (i.e., a condom, a diaphragm, or a sponge) or an intrauterine device (IUD). The later contraceptive devicesinclude the classical IUD, copper contacting IUD, as well as the newer levonorgestrel-releasing IUD(23).

Hormonal contraception, on the other hand, can also promote the elimination of certain ASMs. Specifically, these are ASMs that are metabolized by the UGT enzymes. For example, lamotrigine levels are decreased by about 50% by combined OCPs within one week of co-administration [38,39,40,41]. Valproic acid levels are also significantly lowered by hormonal contraception [27]. The reduction in both of these medications’ levels is due to the ethinylestradiol component of OCPs [24,41,42]. One study also suggested that progestins (drospirenone and levonorgestrel) in combined OCPs reduced serum concentrations of lamotrigine in WWE [43]. If OCPs are indicated in WWE who are on these ASMs, it important to follow ASMs levels closely and adjust the dosages as needed to match the ASMs’ levels from prior to initiation of OCPs.

## 6. Appropriate Contraception during Reproductive Age

Selecting appropriate contraception is important in WWE with reproductive potential due to their higher risk of unintended pregnancies when compared with women without epilepsy [44]. A retrospective survey between 2010 and 2014 by the Epilepsy Birth Control Registry (EBCR) reported that 65% of pregnancies in WWE were unplanned. Of those unplanned pregnancies, 34.7% occurred in WWE who were not on contraception [44]. The EBCR also reported 69.7% of the WWE at risk of unintended pregnancy used highly effective contraception (hormonal, IUD, tubal, vasectomy) [44]. Of the 87.2% that are followed by a neurologist, only 25.4% were consulted about contraception [44]. Furthermore, a retrospective study found that WWE who received IUD-specific counseling by their neurologist were significantly more likely to switch to an IUD (44.4%), compared with women who received no contraceptive counseling (6.5%) [45]. Thus, neurologists can play important roles in helping WWE choose appropriate contraception.

Since enzyme-inducing ASMs can make hormonal contraceptives less effective, patients need to consider a barrier method of contraception (vaginal ring, condom) as an additional method besides OCPs or consider long-acting forms of contraception such as an intrauterine device. Progestin-containing IUDs are safe and acceptable long-acting contraceptives for WWE, as enzyme-inducing ASMs are not known to alter their efficacy [46,47]. A copper IUD is also effective, as it does not contain hormones [44,45]. Depot medroxyprogesterone acetate (DMPA) is another long-acting reversible contraceptive that is effective but can be a less favorable option due to its side effect profile [48]. Long-term use of DMPA can reduce bone mineral density and can delay returning to fertility from months to up to one year [48,49,50,51,52]. Some patients may need to shorten the DMPA dosing frequency from the standard 12 weeks to 8–10 weeks when used with enzyme-inducing ASMssuch as clobazam [48]. Ultimately, choosing a contraceptive method should be individualized with guidance from an obstetrician/gynecologist and neurologist.

## 7. Special Considerations for Catamenial Epilepsy

Catamenial epilepsy (CE) affects approximately a third of WWE [1]. CE is considered a form of drug-refractory epilepsy that is clinically diagnosed after reviewing seizure occurrences in two menstrual cycles, usually with an increase in seizure frequency by two-fold or greater during the menstrual cycle [1,53]. These episodes are seen in all types of epilepsy but particularly in temporal lobe epilepsy [54]. Fluctuations in the estrogen and progesterone plasma levels are believed to be responsible for CE. Studies have shown that estrogen induces seizures by impacting glutamatergic transmission and increasing neuronal excitation [55,56]. Progesterone and its metabolite allopregnanolone rapidly exert anti-convulsant action by binding to progesterone receptors to modulate gamma-aminobutyric acid (GABA-A) receptor expression and by allosterically potentiating GABA-A receptors to induce inhibitory effects, respectively [55,57].

The menstrual cycle can vary from one woman to another but typically occurs every 28 days and can be divided into two phases: (1) follicular phase, which is the first day of menses until ovulation (day +1 to 14), and (2) luteal phase, which is from ovulation to menstruation (day −14 to 1+). Correlating the marked fluctuations of estrogen and progesterone levels and seizure frequency led to the recognition of three CE patterns (Table 1). Premenstrual pattern (C1) occurs during progesterone withdrawal, from days −3 to 3+, which causes an increase in seizure activity before, during, or after the onset of menstruation [1,53]. Periovulatory pattern (C2) occurs from days +10 to −13 due to the sudden increase in estrogen levels during the ovulatory phase [1,53]. Luteal phase pattern (C3) occurs from day −14 to +1 when the estrogen/progesterone ratio is elevated or progesterone levels do not sufficiently rise [1,53]. The C3 pattern is more commonly seen in women who do not ovulate. Based on the National Institutes of Health (NIH) Progesterone Treatment Trial, the prevalence of CE by pattern was 39.8% for C1, 33.9% for C2, and 47.1% for C3 [58].

At this time, there are no ASMs approved by the Food and Drug Administration for treating CE. The ASM regimen should be optimized by increasing the medications’ dose during the cycle of exacerbation. Administering additional ASMs, such as benzodiazepines (lorazepam, clonazepam, or clobazam) can also be considered. Clobazam has been used as an effective off-label ASM for CE. In a double-blind cross-over study, CE patients treated with 20 to 30 mg of clobazam a day for 10 days around menstruation in successive menstrual cycles had reported a significant reduction in seizure frequency [59]. Treatment with acetazolamide (Diamox) led to a 50% reduction in seizure frequency in 40% of women with premenstrual seizures in a small retrospective study [60].

If non-hormonal treatments fail, adjuvant hormonal therapy can be considered. While the research in this area is limited, hormonal therapy with progesterone in lozenge and suppository forms may provide favorable results. The 2015 NIH Progesterone Trial produced class III evidence for treating C1 pattern CE in patients on a baseline optimal ASM regimen with adjunctive progesterone 200 mg (in a lozenge form) PO three times daily from days 14–25; 100 mg (½ a 200 mg lozenge) PO three times daily on days 26–27; 50 mg (¼ of a 200 mg lozenge) PO three times daily on day 28 with subsequent discontinuation of progesterone until day 14 [58]. Additionally, smaller studies with cyclic progesterone treatment have also shown a decline in seizure frequency [61,62,63]. Side effects of progesterone therapy include sedation, depression, fatigue, weight gain, irregular vaginal bleeding, and constipation [61]. Synthetic neurosteroids, such as ganaxolone (analog of allopregnanolone), lack hormonal side effects and can be taken throughout the cycle, but further clinical studies are needed because limited information is available regarding its use in CE [64].

WWE with CE whose timing of periods is difficult to predict, because of the periods’ irregularity, can be treated with synthetic gonadotropin-releasing hormone (GnRH). This will lead to pharmacologically induced amenorrhea through decreasing luteinizing hormone and estrogen production. In women with refractory premenstrual seizures, gonadotropin-releasing hormone analog provided a significant reduction in seizure frequency [65,66]. Although an exacerbation of seizures was noted during the initial three weeks of GnRH therapy due to an estrogen flare before inhibition [66].

In the Birth Control Registry study, an observational study of 750 women aged 18–47, DMPA decreased seizure frequency to a larger degree than combination OCPs or progestin-only contraceptives [67]. The standard DMPA administration (every 12 weeks) induces amenorrhea 50–60% of women at 1 year of treatment; shorter dosing intervals (e.g., every 8–10 weeks) can be used for treating CE [68].

## 8. Specialized Considerations for Pregnancy

The recommended management of WWE during pregnancy includes ASM monotherapy (or the least amount of possible ASMs to achieve seizure control) at the lowest dose possible and avoiding medication changes or additional ASMs [6,7]. ASM substitution or reduction should occur prior to conception as changing medication during pregnancy can increase the risk of seizure recurrence [6,7].

Physiological changes occurring in pregnancy can affect the pharmacokinetics of various medications. These changes include changes in both renal and hepatic function as well as the alteration in serum albumin concentration. One of these changes is a 40–50% increase in the glomerular filtration rate. This leads to a reduction in the concentration of ASMs that are cleared by the kidneys, such as levetiracetam, gabapentin, pregabalin, topiramate, and zonisamide [3,4,5].

The hepatic changes during pregnancy involve both the cytochrome P450 enzymatic system and glucuronidation. Certain cytochrome P450 enzymes (CYP3A4, CYP2D6, and CYP2C9) can increase the metabolism of ASMs, while others (CYP412A and CYP2C19) can decrease such metabolism [3]. In cases of increased metabolism, ASMs doses may need to be increased during pregnancy to maintain efficacy. Alternatively, for ASMs with decreased metabolism in pregnancy, dose reductions (usually minor) may be necessary to minimize potential toxicity [3].

Glucuronidation significantly increases the metabolism of lamotrigine. Its clearance progressively increases throughout pregnancy, leading to a marked reduction in plasma concentration [69]. The clearance reaches a peak of >330% of baseline by 32 weeks in about 77% of women, while in 23%, the peak clearance is less profound, due to genetic polymorphisms [69]. Postpartum, its clearance returns to baseline within 3 weeks. The potential mechanism of increased lamotrigine clearance is due to the upregulation of the UDP-glucuronosyltransferase (UGT) 1A4 enzyme by 17 beta-estradiol [70]. If patients on lamotrigine are considered “rapid metabolizers” due to genetic polymorphisms of the UGT enzyme, their lamotrigine levels may need to be monitored as frequently as every 2 weeks [71,72,73].

Lastly, maternal albumin alpha-1 acidglycoprotein decreases in pregnancy, resulting in an increase in unbound drug concentration of certain ASMs (phenytoin, valproic acid, carbamazepine, clobazam) [3]. Thus, monthly monitoring of the drugs’ free plasma levels becomes even more essential in patients who are taking ASMs bound to albumin, to prevent toxicity [3].

## 9. Discussion

The hormonal changes in WWE during their reproductive years can affect seizure frequency. For these women, the hormonal influences have the most prominent manifestations at specific time periods of the menstrual cycle (when estrogen concentration is typically increased more than that of progesterone) and in the setting of hormonal contraception. A third of WWE experience catamenial seizures [1]. Estrogen is considered a proconvulsant hormone, while progesterone is considered an anti-convulsant hormone [55,56,57]. Various treatment methods have been derived to help mitigate catamenial seizures. The treatment options include optimizing the current ASM regimen, administering additional ASMs such as benzodiazepines or hormonal therapy (progesterone in lozenge and suppository forms, GnRH agonists, and DMPA), to medically induce amenorrhea [58,59,61,62]. Both seizures and ASMs can negatively impact reproductive health by disturbing ovarian function. Reproductive endocrine disorders are more common in WWE than in the general population [14,15]. For instance, PCOS occurs at a higher rate in WWE than in the general population, particularly with valproic acid use [14].

The interaction between enzyme-inducing ASMs and hormonal contraceptives can lead to failure of either treatment [23]. Enzyme-inducing ASMs can lower serum levels of reproductive steroids, leading to failure of hormonal contraception and unplanned pregnancy. Conversely, hormonal contraceptives can promote the elimination of ASMs metabolized via glucuronidation such as lamotrigine [38,39,40,41]. The increased metabolism can lead to breakthrough seizures. Thus, selecting an optimal contraception method is essential in WWE with reproductive potential, especially given this patient population’s higher risk of unintended pregnancies [44]. The approach to the treatment should be individualized with the assistance of the patient’s neurologist and OB/GYN with the goal of finding an appropriate contraception method that has the least interaction with ASMs [45]. To avoid failure of hormonal contraception due to ASMs, women can opt for non-hormonal methods or consider long-acting forms of contraceptives such as IUDs [47,48]. To avoid breakthrough seizures due to hormonal contraception, doses of the ASMs may need to be increased, or the contraception method may need to be changed. It is important to prescribe proper ASMs and supplement with folic acid before the patient becomes pregnant to reduce teratogenicity and cognitive problems in future offspring [6,7,44].

The physiological changes induced by pregnancy can alter the pharmacokinetics of ASMs. Most notably, upregulation of UTG enzymes due to estrogen’s effect during pregnancy can increase the metabolism of lamotrigine [70]. Therefore, it is necessary to carefully monitor serum ASMs levels throughout pregnancy and adjust dosages as needed to the patient’s prepregnancy baseline to prevent breakthrough seizures.

In summary, the management of epilepsy in women of reproductive age is complicated by the interactions between hormones, seizures, and ASMs. These interactions must be taken into consideration to develop an optimal treatment regimen and prevent both breakthrough seizures and harm to reproductive health.

## Figures and Tables

**Table 1:** List of anti-seizure medications (ASMs) that do and do not affect hormonal contraception through hepatic enzyme induction.

ASMs That Decrease the Effectiveness of Hormonal Contraceptives	ASMs That Do Not Change the Effectiveness of Hormonal Contraceptives
Carbamazepine (Tegretol, Tegretol XR, Carbatrol, Equetri) Phenobarbital Phenytoin (Dilantin) Primidone (Mysoline) Clobazam (Onfi) Felbamate (Felbatol) Eslicarbazepine (Aptiom) Lamotrigine (Lamictal) ^1^ Oxcarbazepine (Trileptal) Preampanel ^2^ Rufinamide (Banzel) Topiramate (Topamax) ^3^	Acetazolamide (Diamox) Clonazepam (Klonopin) Diazepam (Valium) Valproic Acid (depakote, Depakene) Ethosuximide (Zarontin) Gabapentin (Neurotin, Graslise, Horizant) Lacosamide (Vimpat) Levetiracetam (Keppra) Lorazepam (Ativan) Pregabalin (Lyrica) Tigabine (Gabitril) Vigabatrin (Sabril) Zonisamide (Zonegran)

^1^. Lamictal is a weak inducer, at doses of 300 mg daily or more. ^2^. Perampanel is a weak inducer at doses 12 mg or higher. ^3^. Topiramate induces hormonal contraceptives at doses of 200 mg daily or higher. Adapted from Reddy, D.S. Clinical Pharmacokinetic Interactions between Antiepileptic Drugs and Hormonal Contraceptives. *Expert Review of Clinical Pharmacology***2010**, *3*, 183–192, doi:10.1586/ecp.10.3.
